# Left-side incarcerated Amyand’s hernia with appendix and caecum provoke by early banana diet: a case report

**DOI:** 10.1186/s12876-021-01752-2

**Published:** 2021-04-13

**Authors:** Supangat, Henggar Allest Pratama, Nanda Eka Sri Sejati, Brenda Desy Romadhon, Ina Sulistyani

**Affiliations:** 1 Pharmacology Department, Faculty of Medicine, Jember University, Jember, 68121 Indonesia; 2 Molecular Biology Research Center, Jember University, Jember, 68121 Indonesia; 3 Department of Pediatric Surgery, Dr. Soebandi Hospital, Jember, 68111 Indonesia; 4 Emergency Department, Dr. Soebandi Hospital, Jember, 68111 Indonesia; 5 Radiology Deparment, Dr. Koesnadi Hospital, Bondowoso, 68214 Indonesia

**Keywords:** Amyand’s hernia, Banana early feeding, Symptoms of intestinal obstruction

## Abstract

**Background:**

Amyand’s hernia was an unusual condition defined by the presence of an appendix located in the inguinal hernia sac. Its prevalence was 1% of all inguinal hernia in children. The clinical manifestation of Amyand’s hernia was depending on hernia’s stage, an incarcerated hernia will present with an inguinal mass following by pain and motility disorder. It could lead to abdominal distention in the late stage. The common location of Amyand’s hernia was on the right side, the left side was uncommon. Early feeding on infants could provoke symptoms of bowel obstruction (SBO). More than 76% of infants in Java, Indonesia was given banana as solid food in infants before six months old. There is a correlation between the early banana diet and SBO. Amyand’s hernia could present as morbidity of early banana diet.

**Case presentation:**

We describe a case of two months old infant present with an incarcerated left inguinal hernia and history of early banana diet that performed herniotomy procedure. During the operation, we found left-side incarcerated Amyand’s hernia with appendicitis, excoriation caecum, and sticky banana mass.

**Conclusion:**

This case suggest the possibility of early feeding of banana diet may provoke incarceration of an inguinal hernia and if the incarcerated hernia content contains the appendix, then an Amyand's hernia.

## Background

The inguinal hernia was defined as a protrusion of fascia or visceral organ through the abdominal wall into the inguinal canal. In children, an inguinal hernia is caused by obliteration failure of processus vaginalis during gonad development [1]. Rarely, the appendix can be found in the inguinal sac of an inguinal hernia which has been called an Amyand's hernia. Amyand’s hernia was rare. The most common of Amyand’s hernia was present in elder patients (over 70 years) and children, although the prevalence in children might reach 1% of all inguinal hernia [2]. Appendicitis could accompany Amyand’s hernia incidence. The incidence of Amyand’s hernia associated with appendicitis was even rarer. It’s estimated only 0.07–0.13% [3].

The clinical manifestation of Amyand’s hernia was difficult to differentiate with another inguinal hernia. In the incarceration stage, it’s common to present with an inguinal mass following by pain and motility disorder. In the late stage could lead to abdominal distention as symptoms of bowel obstruction (SBO). Amyand’s hernia was common to present on the right side based on the appendix vermiform position but in a rare condition, it could find in the left-side. Left-side Amyand’s hernia could be associated with situs inversus, intestinal malrotation, and mobile caecum [4].

The early banana diet is very common in Indonesia, especially in Java. More than 76% of infants in Java, Indonesia is given banana as solid food in infants before six months old. There is a correlation between the early banana diet and SBO [5].

## Case presentation

A 2 months old male infant was taken to Emergency Department Soebandi Hospital with abdominal distention and bilious vomiting. The symptoms were present after the left inguinal hernia appear and could not be reduced for 12 h before. A left inguinal hernia was present from two weeks old and could be reduced spontaneously before. An early banana diet was given to the patient one day before the symptoms appear. No other food was given except breast milk.

**Fig. 1:**
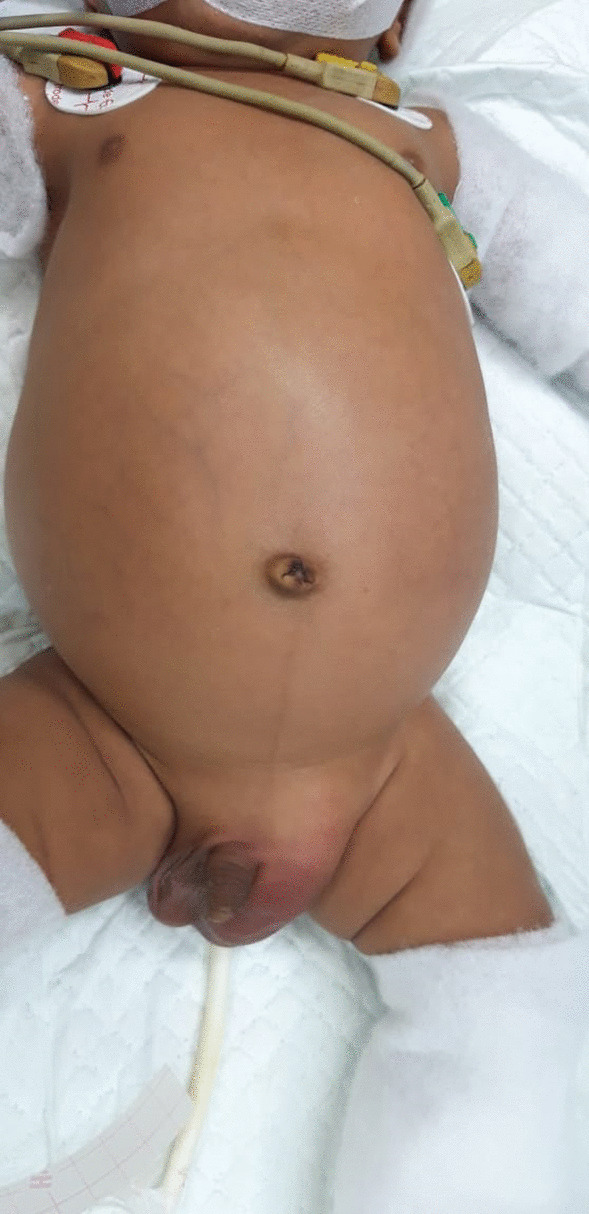
Physical examination of the patient. This is the first impression of patients in the Emergency Department. We can see abdominal distention and left inguinal hernia

**Fig. 2:**
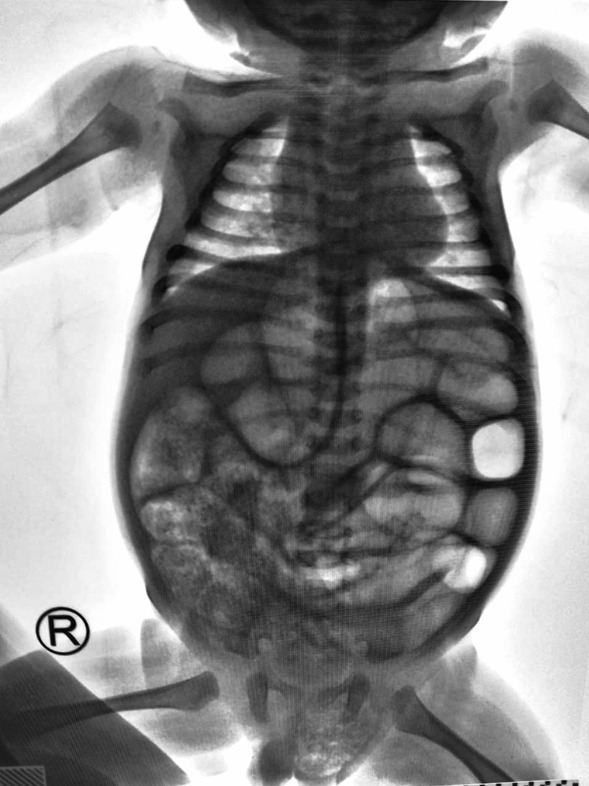
X-ray’s examination. This is the X-ray examination in the Emergency Department. We can see obstructed bowel and caecum in the left inguinal hernia

From the physical examination, we found abdominal distention with hypertympanic and increasing bowel sound as a sign of intestinal obstruction (Fig. 1). We also found a sign of dehydration. Radiology examination found an obstructed bowel with caecum visualized at left inguinal hernia (Fig. 2).

**Fig. 3:**
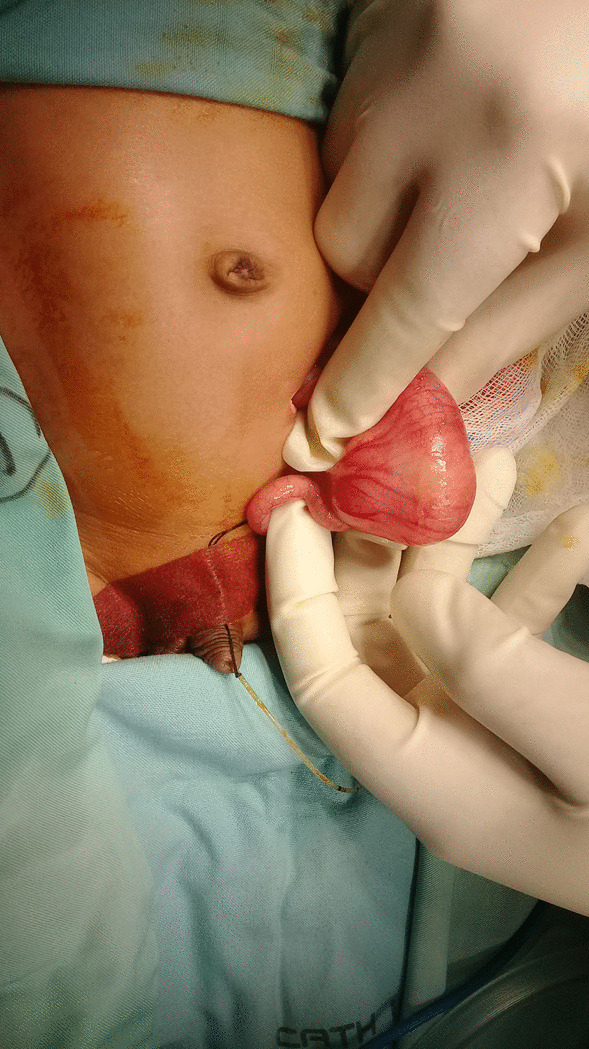
Durante op finding. We found left-side Amyand’s hernia with appendicitis and caecum. We also found a sticky banana mass in the caecum

Fluid resuscitation and nasogastric tube decompression were performing followed by broad-spectrum antibiotics. Then the patient planned to a herniotomy. During the operation, we found left-side incarcerated Amyand’s hernia with appendicitis and erythematous caecum. No perforation was performed. We also found sticky banana mass to support the suggestion of early banana diet as a predisposing factor (Fig. 3.).

Then we perform an appendectomy, reposition of the caecum to the abdomen, and ligation of the sac. It has a good result. There was no postoperative complication. It has a good functional bowel and the patient could take oral breast milk gradually increase. The patient’s parent was satisfied with this treatment.

## Discussion and conclusion

Amyand’s hernia was not easily diagnosed pre-operatively. It was difficult to differentiate with another inguinal hernia. The patient could come to the Emergency Department with inguinal mass associated with pain and SBO or inguinoscrotal swelling as a sign and symptom of an incarcerated hernia. Fever could present at appendicitis but not always present. At late presentation, abdominal distention and vomiting could present. It depends on the condition of appendix vermiform whether inflammation or perforated [6]. Radiological X-ray could notice bowel dilatation. Ultrasound and abdominal CT could help to diagnosed hernia but clinical symptoms were very important to measure the stage and the next procedure performs [7].

Appendicitis could perform following Amyand’s hernia. The possibility caused was obstruction of incarcerated hernia restricts appendix blood flow and bacterial growth lead to inflammation and perforation. Adhesion and obstruction of the hernia neck caused the appendix trapped in the hernia sac [8]. Classification of Amyand’s hernia was depending on appendix vermiform condition. Type I is normal appendix within an inguinal hernia. Type II is acute appendicitis within an inguinal hernia, no abdominal sepsis. Type III is acute appendicitis within an inguinal hernia, abdominal wall, or peritoneal sepsis. Type IV is acute appendicitis within an inguinal hernia, related or unrelated abdominal pathology. This classification will determine the next procedure. Type II Amyand’s hernia classification is based on inflammation of appendix vermiform without perforate and abdominal sepsis. Appendectomy and ligation of the sac have been recommended to treat type II Amyand’s hernia [9].

The right-side inguinal hernia was the most common condition of Amyand’s hernia. Left-side was uncommon. Cigsar et al. reported from 11 years of experience to treat Amyand’s hernia, left-side Amyand’s hernia occurs in 4.3%. It could be associated with situs inversus, intestinal malrotation, and mobile caecum. Mobile caecum was the only finding based on the research while situs inversus and intestinal malrotation was still a suggestion based on theory [10]. In oyr patient, mobile caecum was found.

The early banana diet was very popular in Indonesia. More than 74% infant was given by early banana diet as solid food before six months old. The culture associated and lack awareness of potential danger has been suggested as a predisposing factor of early banana diet. An early banana diet could induce symptoms of bowel obstruction (SBO). Wiryo et al. reported from 3420 neonates in Indonesia, the relative risk of early banana diet that provokes SBO was 9.18. It was concluded that an early banana diet was an important risk factor of SBO [11]. The carbohydrate crystal line pattern (b type) and the non starch polysaccharide content found in banana such as hemicellulose, algine, and pectin are material that are difficult to digest. Therefore, they are potential for fermentation and gas production. The gas could cause abdominal distention and vomiting. Banana as a solid food may get stuck at the narrowing bowel. An inguinal hernia could narrow the bowel so solid food could get stuck and provoke incarceration [12].

Amyand’s hernia was a rare condition of inguinal hernia and left-side Amyand’s hernia was even rarer. The early banana diet was very common in Indonesia. It could provoke the incarceration of an inguinal hernia that was presented before. This case suggest the possibility of early feeding of banana diet may provoke incarceration of an inguinal hernia and if the incarcerated hernia content contains the appendix, then an Amyand's hernia. This case could be a good example to educate the mothers in Indonesia to prevent giving early banana diet in infants. Therefore, it was necessary to educating not giving an early banana diet to prevent intestinal obstruction in infants.

## Data Availability

All information of the patient came from the Department of Surgery and Department of Pediatric Soebandi Hospital, Jember, Indonesia.

## Abbreviation

SBO: Sign of bowel obstruction
